# Nonpalpable breast masses: One-year ultrasound follow-up and morbidity outcomes in Shanghai

**DOI:** 10.1097/MD.0000000000023556

**Published:** 2020-12-11

**Authors:** Hongmei Wen, Tao Xu, Qinhua Huang, Chumiao Zhang, Qi Zhang, Haiyan Chen

**Affiliations:** a Department of General Practice, Zhangyan Community Healthcare Center; b Department of Health Check-Up Center, Jinshan Hospital, Fudan University, Shanghai, China.

**Keywords:** breast cancer, cancer morbidity, cancer screening, early diagnosis, nonpalpable breast mass

## Abstract

Women with nonpalpable breast masses are at a high risk of developing breast cancer (BC) due to misdiagnosis during the follow-up period.

A total of 40,334 women were divided into palpable and nonpalpable breast mass groups. We assessed the risk factors for cancer development in patients with nonpalpable breast masses during a 1-year follow-up period.

Of the 1335 patients in the nonpalpable breast mass group, we found 50 patients of BC, of which 35 patients accepted surgery and were confirmed with biopsy at the beginning of the study. The remaining 15 (1.1%) were diagnosed with BC during follow-up, and included 10 *in situ* and 5 invasive carcinomas. Four of the 10 patients in the *in situ* subgroup, and 2 out of the 5 in the invasive subgroup were overweight (Body mass index > 24 kg/m^2^). Nine in situ BC patients had breast-conserving surgery, 1 had a mastectomy. No patient in the *in situ* group received chemotherapy or radiotherapy. All 5 patients with invasive disease received 6 cycles of chemotherapy. Only 3 (20%) of the 15 patients with BC had a positive family history. We found 131 BC cases, including BC detected during screening (81) and follow-up (50). The incidence of BC was 240.2 per 100,000 inhabitants.

Patients with nonpalpable breast masses require regular follow-up as they have a high risk of cancer occurrence. Regular follow-up can lead to early diagnosis and effective treatment of these early-stage BC patients.

## 1 Introduction

Globally, breast cancer (BC) is the most common malignancy in women; in China alone, 169,000 cases are reported every year, with a mortality rate close to 26%.^[1]^ In the past 20 years, BC morbidity has increased by 30% to 40% in many countries.^[2]^ The BC morbidity rate in China increases by approximately 2.9% every year and remains higher than in American white women (1.1%) and Asian women in general (1.5%).^[3]^

Early diagnosis of BC is key to improving survival and quality of life. A trend towards decreased BC-associated mortality has recently been observed in the UK and USA, owing to screening and early diagnosis.^[4,5]^ In developed countries, 1 out of 3 patients diagnosed with early-stage BC have 5 and 10 years survival rates of 97% and 94% respectively, and overall BC mortality has decreased by 30% to 50%.^[9,10,11]^ But in China, stage-I BC is diagnosed less commonly, at only 10% in Shanghai and even lower rates in other regions. Advanced stage diagnosis is very common in China, leading to poor survival and quality of life.^[6]^*In situ* breast carcinoma has absolute curable rates, while stages I, II, and III have 5-year survival rates of 97%, 75%, and 45%, respectively.^[7]^

We evaluated the risk of cancer in breast nodules using the Breast Imaging Reporting and Data System (BI-RADS), a scale commonly used with ultrasound, mammography, and magnetic resonance imaging (MRI). The BI-RADS scores breast nodules from 0 to 6, and the risk of malignancy increases with an increased score. Breast nodules are not specifically defined; palpable breast masses are termed breast masses, while nonpalpable ones identified by imaging are referred to as breast nodules.^[8]^ A nonpalpable breast mass is 1 that cannot be found during clinical examination of the breast, but can be identified by ultrasound, mammography, and MRI; a mass diagnosed as cancer is termed nonpalpable BC.^[23]^ In 20% to 30% of patients with nonpalpable breast masses, breast nodules develop into cancer.

We adopted ultrasound during follow-up visits due to its repeatability, the high density of breast tissue in Chinese women, and because most Chinese women are affected before 50 years of age — 10 years earlier than Western women.^[7]^ The sensitivity and specificity of mammography remains suboptimal in Chinese patients due to dense breast tissue. A clinical breast examination protocol for nonpalpable masses has been recommended by some studies to improve early diagnosis of BC, although current global guidelines recommend mammography for screening.^[12]^ The sensitivity of mammography-based diagnosis is lower in Chinese women, and the Chinese Anti-cancer Association recommends that an ultrasound scan can be used as a supplementary method in BC screening.^[13]^

Previously published studies indicate that overweight/obesity can contribute to the risk of BC. In a previous cohort study, International Agency for Research on Cancer observed that BC is mildly associated with overweight and obesity, with odds ratios (ORs)< 2.0.^[14]^ A meta-analysis of 31 studies revealed that an increase in the body mass index (BMI) by 5 kg/m^2^ increases the probability of BC occurrence by 12%.^[2,15]^ Another study concluded that the risk of BC in overweight/obese women is 6.78 times higher compared with normal-weight postmenopausal women.^[16]^ Overweight/obesity induces endocrine dyscrasia, which may be 1 of the main causes of increased risk of BC.^[17]^ Also, obesity induces lipid solubility in the body and induces BC.^[18]^ Other studies have shown that obese patients tend to have larger tumors compared with their normal- weight counterparts, indicating a role for obesity in the prognosis of BC.^[19,21]^ Genetic factors and family history also play important roles in the risk of BC.^[20,22]^

Numerous genetic, lifestyle and environmental factors affect the risk of BC.^[24]^ Among these, genetic factors are of particular importance.^[25]^ Studies have found that strong family history can quadruple a woman's risk of BC.^[26]^

In this study, we compared patients with palpable and nonpalpable breast masses for their BMI values, positive family history, and cancer morbidity rates. We explored whether patients with nonpalpable breast masses required surgical intervention to prevent cancer development at a later stage, and evaluated the predictors of BC in patients with nonpalpable breast masses.

## 2 Materials and methods

The local government of Shanghai provided funds to screen adult female residents for BC every 2 years, and more than 50000 women were sent to Jinshan Hospital, Fudan University. They were either residents of Shanghai or had lived there for more than 5 years. The study protocol was approved by the Hospital ethics committee. Two specialized surgeons and 2 ultrasound specialists, each with more than 20 years of experience, performed clinical examinations and breast ultrasounds on the included patients.

Patients came for a follow-up to our center every 3 months for 1 year. Some women with breast masses believed that their mass, being nonpalpable, did not need follow-up and tended to ignore follow-up visits. To assess the morbidity in this group and to ensure follow-up, our strategies were to call them, pay for their transportation, and offer free examination.

Included patients were divided into 2 groups: the palpable and nonpalpable breast mass groups. Ultrasound images were scaled using the BI-RADS score, and patients who scored ≥4 were advised to undergo surgery. All patients with scores of 5 and 6 consented to surgery, and patients who refused surgery were monitored for 1 year with biopsy. All patients with palpable masses were advised surgery regardless of their BI-RADS scores, and most of them underwent surgery. These patients with palpable masses had BI-RADS scores between 1 and 4. Although many patients in the palpable group were at undue risk of developing BC, we recommended surgery to all to rule out the risk of missing BC diagnosis due to loss to follow-up.

Patients with nonpalpable breast masses were followed up for 1 year (1 January to 31 December 2016) and assessed for BMI, positive family history, and development of BC. The BMI values into 2 groups: < 24 kg/m^2^ and ≥24 kg/m^2^.

Nonpalpable breast masses were defined when both senior surgeons could not palpate the masses that both ultrasound specialists had identified on ultrasound. These masses were either less than 1 cm, or in deep positions, or these patients had large breasts and the senior surgeons were unable to palpate these masses. Masses < 1 cm positioned near the surface in patients with relatively small breasts, could still be palpated by both surgeons. We compared the BC diagnosis rates between the nonpalpable and palpable mass groups.

The positive family history criteria were: 1 or more direct and (or) second degree female relative diagnosed with breast or ovarian cancer; 2 or more third degree female relatives diagnosed with breast or ovarian cancer. To assess the family history, the guidelines and standards of BC diagnosis and treatment (version 2015) of the Chinese Anti-cancer Association were used.^[14]^

### 2.1 Statistical analysis

Data were analyzed using the SPSS 22.0 software (IBM, Armonk, NY). Continuous variables were expressed as mean ± standard deviation (SD), and categorical variables were expressed as frequency and percentage. The normality of data distribution for age, BMI, length and width of the mass, and estrogen level (continuous variables) was tested using the Kolmogorov-Smirnov test. In the absence of normal distribution, these continuous variables were compared between the groups using the Mann-Whitney test. Positive family history, postmenopausal status, and the presence or absence of BC (categorical variables) were compared between the groups using the chi-square test.

Binomial logistic regression analysis was applied to the subset of patients with available biopsy diagnosis of malignant or benign lesions. The presence of BC was selected as a dependent variable, and the age, BMI, size of the mass, positive family history, menopausal status, and palpability of the mass were the predictors considered in the regression model. Patients in the nonpalpable subgroup whose biopsy diagnosis was available were selected, and logistic regression was conducted using age, BMI, length of the mass, positive family history, and menopausal status as predictor variables for the presence of BC. In the same dataset, receiver operating characteristic (ROC) analysis was performed for estrogen levels and continuous variables shown to be significant predictors of BC to determine the cut-off values. All analyses were performed at a 2-tailed 0.05 significance level.

## 3 Results

**Figure 1:**
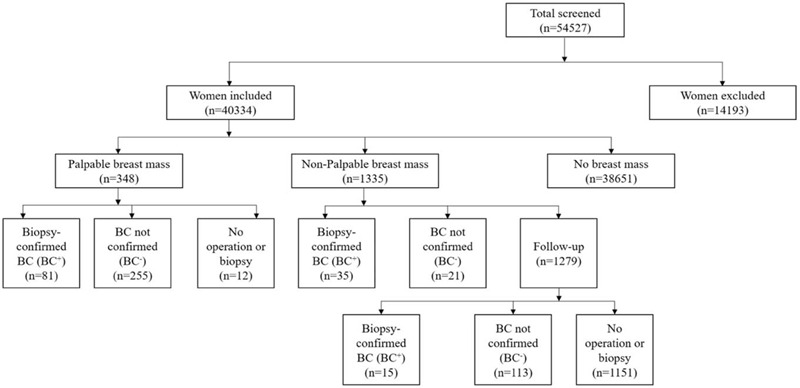
Study patient flow chart.

Out of the 54527 women (age 18–70 years) undergoing a physical examination at Jinshan Hospital from 1 January 2014 to 31 December 2015, 40334 women (mean age 38.9 ± 10.9 years) were included in this study. A total of 14193 patients, excluded from the follow-up schedule, were those who refused to complete the questionnaire, refused breast ultrasound, refused to attend the follow-up plan, did not follow-up regularly, or had incomplete data or no contact information. 40334 women included in this study and underwent clinical breast examination and breast ultrasound, 116 were diagnosed with BC and underwent surgery with postoperative biopsy. Among them, 35 women had clinically nonpalpable tumors and 81 had palpable tumors. In the remaining patients with nonpalpable masses, 15 more BC patients were identified during the 1-year follow-up. Overall, 336 women (0.83%) had palpable, and 1335 women (3.3%) had nonpalpable breast masses, as shown in Figure 1. The calculated morbidity ratio = 81 + 35 + 15/14193 + 40334 = 240.2/100,000

### 3.1 Characteristics of patients with a palpable breast mass

In the palpable breast mass subgroup, a positive family history of BC was observed in 41.1%, a total of 76.5% were premenopausal, and the average mass size was 11.9 ± 2.5 mm (Table 1). All patients from the palpable mass group were recommended surgery, and within 3 months 336 of 348 had undergone breast surgery (Table 2). In the operated group, a biopsy-confirmed invasive BC in 81 (24.1%) patients and the absence of BC in the remaining 255 patients.

**Table 1:** Comparison of main demographic, clinical, and pathological data between the group with palpable mass and nonpalpable breast mass.

	Palpable mass	Nonpalpable mass	*P*-value
Mean age [yr]	49.15 (8.58)	41.11 (11.44)	<.001^a^ (U = 129,591)
Mean BMI [kg/m^2^]	24.19 (2.27)	22.69 (2.87)	<.001^a^ (U = 147,375)
Mean length of mass [mm]	11.91 (2.51)	6.32 (1.79)	<.001^a^ (U = 6,868)
Mean width of mass [mm]	9.62 (2.19)	4.27 (1.35)	<.001^a^ (U = 5,552)
Mean estrogen level [pmol/L]	161.63 (79.90)	198.88 (36.66)	<.001^a^ (U = 181,307.5)
Positive family history (N,%)	138 (41.07)	315 (23.60)	<.001^b^ (Chi-square= 41.5, df = 1)
Postmenopausal status (N,%)	79 (23.51)	155 (11.61)	<.001^b^ (Chi-square= 31.6, df = 1)
BC detected (N,% of those with biopsy)	81 (24.11)	50 (27.17)	.441^b^ (Chi-square= 0.593, df = 1)
BC detected (N,% of total group size)	81 (24.11)	50 (3.75)	<.001^b^ (Chi-square= 154, df = 1)

Continuous variables are shown as mean (SD) and categorical variables as number (%).

a Mann-Whitney test.

b Chi-square test, BC = breast cancer.

**Table 2:** Distribution of BC among the palpable and nonpalpable mass groups: demographic, clinical, and pathological characteristics of patients.

	PALPABLE				NONPALPABLE									
	From screening				From screening				From follow up				No operation/	
	BC + (N = 81)		BC− (N = 255)		BC + (N = 35)		BC− (N = 21)		BC + (N = 15)		BC− (N = 113)		No biopsy (N = 1151)	
	Mean	SD	Mean	SD	Mean	SD	Mean	SD	Mean	SD	Mean	SD	Mean	SD
Age [yr]	47.05	5.83	49.82	9.19	52.49	9.2	38.05	10.37	49.2	3.14	39.06	8.3	40.91	11.63
BMI [kg/m^2^]	23.99	2.09	24.26	2.33	22.79	3.26	22.22	2.96	22.9	2.18	23.25	2.63	22.63	2.89
Length of the mass [mm]	11.75	2.08	11.96	2.64	6.51	1.92	8.76	0.44	6	1.6	7.04	1.97	6.21	1.74
Width of the mass [mm]	9.77	1.78	9.57	2.3	4.11	1.28	6.05	1.12	4.07	1.28	4.64	1.44	4.21	1.32
Estrogen level [pmol/L]	21.22	1.75	206.23	11.63	23.97	2.99	205.52	14.83	21.87	2	207.96	12.12	205.49	12.18
Positive family history [N,%]	47	58	91	35.7	15	42.9	6	28.6	3	20	15	13.3	276	24
Postmenopause [N,%]	6	7.4	73	28.6	18	51.4	1	4.8	3	20	0	0	133	11.6

Continuous data are shown as mean (SD) and categorical as number (%). BC+ = Histologically confirmed breast cancer, BC− = Histologically confirmed that the mass is not a BC, BC** = **breast cancer.

### 3.2 Characteristics of patients with a nonpalpable breast mass

In the nonpalpable breast mass group, less than 25% had a positive family history, almost 90% were premenopausal (88.4%), and the average mass length was 6.3 ± 1.8 mm (Table 1). At the 3-month landmark, 35 (2.6%) of the nonpalpable group had been diagnosed with BC by biopsy. During the 1-year follow-up, tissue specimens were obtained from 128 patients with nonpalpable masses and 15 more cases of BC were diagnosed (Fig. 1, Table 2). Out of these 15 BC cases, 6 (40%) were 50 years or older; BMI values were higher than 24 kg/m^2^ in 4 patients (26.7%); 3 patients (20%) had a positive family history of BC; 12 patients (80%) were premenopausal, and average length of the mass was 6 ± 1.6 mm. Out of these 15 BC cases, 10 (66.7%) were *in situ*, and 5 were invasive.

Three patients (20%) had masses that grew rapidly from nonpalpable to palpable within 1-year, with lengths of approximately 1 cm. The BI-RADS scores of these 3 patients had increased from 3 to 4B, 4A to 4C, and 3 to 4C, respectively. In the remaining 12 patients, the masses had remained nonpalpable, with only a few calcification points seen on ultrasound. All 15 patients were advised to undergo further examination or surgery. Approximately half of them accepted surgery upfront, and postoperative biopsy supported possible malignancy; the other half underwent mammography, the results of which suggested malignancy and they finally accepted the surgical option.

All 15 BC cases were confirmed as early-stage disease with biopsy, and were given the option of breast preservation or reconstruction. No patient accepted breast reconstruction, due to the fear of cancer recurrence. Four out of the 10 patients with *in situ* carcinomas were overweight/obese; 2 out of the 5 invasive carcinoma patients were overweight/obese.

Out of the 113 patients diagnosed as no-BC by biopsy during the follow-up period, 23 (20.4%) patients were aged 50 years or older; 35 (31%) patients were obese (BMI> 24 kg/m^2^); 15 (13.3%) had a positive family history; all 113 were premenopausal, and their average mass sizes were 7.0 ± 2.0 mm.

### 3.3 Comparison of palpable vs. nonpalpable groups

Patients with palpable and nonpalpable masses differed significantly in various parameters (Table 1). The Mann-Whitney *U* values show that patients with palpable masses were older (*P* < .001), had higher BMI (*P* < .001), had greater length and width of the mass (*P* < .001 for both), and lower estrogen levels (*P* < .001), than those with nonpalpable masses. The chi-square and degrees of freedom (df) values suggest that patients with palpable masses had a higher rate of positive family history (*P* < .001), and were more often in the postmenopausal period (*P* < .001). Although the palpable group showed a significantly higher proportion of BC (*P* < .001), the proportion of biopsy-confirmed BC in the operated subgroup was similar to that in the nonpalpable group (*P* = .441).

### 3.4 BC prediction

We detected 131 cases of BC (81 from the palpable mass group and 35 + 15 from the nonpalpable mass group). Univariate analyses revealed that BC positive patients had a higher mean age (*P* = .017) and lower estrogen levels (*P* < .001) than biopsy-confirmed BC negative patients. Although there was a tendency towards lower BMI and length of the mass in BC negative patients, the statistical significance was not reached (*P* = .097; *P* = .093, respectively). In multivariate analyses, binomial logistic regression analysis was used to determine the predictors of biopsy-confirmed BC in the entire sample of patients whose tissue samples were assessed after surgery (n = 520). Advanced age and positive family history were positive predictors for the development of BC (*P* < .001 for both), whereas the length of the mass (*P* = .014) and menopausal status (*P* = .036) were negative predictors. The palpability of mass and BMI were not significant predictors for the development of BC (*P* = .869, *P* = .317, respectively). Though this model was significant (*P* < .001), it could correctly classify those without BC in 96.4% of cases, in contrast to only 8.4% of those with BC.

As there were significant differences in the parameters between the palpable and nonpalpable mass groups, we conducted binomial regression analysis separately in individuals with nonpalpable breast mass whose tissue specimens were obtained (n = 184). Our results showed that the odds for BC could be predicted by age, length of the mass, positive family history, and menopausal status, while BMI was not a significant predictor (Table 3). The odds of developing BC increased with age (OR 1.242 [1.114 – 1.386]), positive family history (OR 18.942 [5.467 – 65.626]) and postmenopausal status (OR 18.7 [1.718 – 203.568]). The length of the mass decreased the OR for BC (OR 0.635 [0.468 – 0.861]). The current model was notably improved (*P* < .001, Negelkerke R square = 0.690), and it could correctly classify 68% of patients with BC and 96.3% of patients without BC.

**Table 3:** Binomial logistic regression analysis: predictors of histologically confirmed breast cancer in patients with nonpalpable breast mass.

							95% CI for OR	
Predictors included in the model	B	S.E.	Wald	df	*P*-value	Odds Ratio (OR)	Lower	Upper
Age	0.217	0.056	15.137	1.000	.000^∗^	1.242	1.114	1.386
BMI	0.233	0.124	3.520	1.000	.061	1.262	0.990	1.610
Length of mass	-0.455	0.156	8.541	1.000	.003^∗^	0.635	0.468	0.861
Family history (Positive)^†^	2.941	0.634	21.524	1.000	.000^∗^	18.942	5.467	65.626
Menopause status (Postmenopausal)^‡^	2.929	1.218	5.780	1.000	.016^∗^	18.700	1.718	203.568
Constant	-14.045	4.639	9.165	1.000	.002	0.000		

CI = confidence interval, df = degrees of freedom, OR, SE = standard error.

∗ p<0.05.

† reference category: negative.

‡ reference category: premenopausal. BMI** = **body mass index.

ROC analysis for age showed area under the curve (AUC) of 0.837 (95% CI: 0.779 – 0.896; *P* < .001) and the cut-off value for BC as 46.5 years (sensitivity 0.78 and specificity 0.72). ROC analysis for the length of the mass showed AUC of 0.641 (95% CI 0.554 – 0.728; *P* = .003) and the cut-off value for BC as 7.5 mm (sensitivity 0.74 and specificity 0.55). ROC analysis for the estrogen level showed AUC of 1 and cut-off value for BC as 106.5 pmol/L (both sensitivity and specificity of 1).

## 4 Discussion

In this study, we evaluated patients with nonpalpable breast masses for age, BMI values, positive family history, the length of the mass and cancer morbidity rates. We asked whether patients with nonpalpable breast masses require surgical intervention to prevent cancer development at a later stage.

Our results indicate that patients with nonpalpable breast masses require careful follow-up, especially if they are elderly, postmenopausal, with a positive family history, or a smaller mass length. These women are at a higher risk of developing BC and can benefit substantially from hormonal, surgical, and lifestyle interventions that reduce this risk.^[27,28]^ Additionally, 25 in situ cancers in patients with nonpalpable breast masses were detected early, in stage-I disease, and therefore had a good prognosis.

Our first finding that BMI > 24 kg/m^2^ in nonpalpable breast mass patients correlates with cancer development shows that obesity/overweight puts these patients at higher risk of cancer development during the following year. ROC analysis in patients with nonpalpable breast mass showed that estrogen levels below 106.5 pmol/L favor BC with maximum sensitivity and specificity; age over 46.5 years may predispose to BC; and the length of the mass < 7.5 mm showed a milder correlation with development of BC.

Our second finding that positive family history in nonpalpable breast mass patients correlates with BC development in the 1-year follow-up period shows that such family history requires careful follow-up, with surgical intervention when BC is diagnosed. Previous studies have also found that strong family history can quadruple a woman's risk of BC.^[26]^ Positive family history was defined as the occurrence of breast or other gynecological cancer in a first-degree relative (sister, mother or daughter), in more than 2 female relatives, or other third female relatives Guidelines and Norms for the Diagnosis and Treatment of BC by the Chinese Anti-cancer Association, version 2015).

Our results show that 12 of the 15 BC patients in the originally nonpalpable group were premenopausal (between 40 and 55 years). This correlates with previous reports that Chinese women manifest BC at a younger age compared with Western women.^[7]^

Our findings indicate that BC cases diagnosed from the nonpalpable breast mass group have an early-stage disease with a good prognosis. All 25 in situ cancer patients were from the nonpalpable group; 15 had BI-RADS scores ≥5 and were operated after an ultrasound, while 10 were detected during the 1-year follow-up period. Of the 81 patients with palpable breast masses, 33 had stage-I and -II disease, indicating an early cancer detection rate of 40.7%, which was lower than that in the nonpalpable group.

Due to enhanced public awareness, advances in breast imaging, and emphasis on early BC detection and prevention, identification of women at high risk of developing BC has gained importance. Fine needle aspiration biopsy, nipple aspirate fluid, and ductal lavage are the 3 minimally invasive procedures used to sample breast tissues in asymptomatic high- risk individuals. Even though they are minimally invasive, many asymptomatic women do not accept these procedures. We used ultrasound as it is noninvasive, acceptable, easy, and repeatable.

In our study, BMI was not significantly associated with the odds of having confirmed BC, which is in disagreement with previously published reports. However, considering that the *P*-value was .061 (close to .05), it is likely that this discrepancy is due to the relatively small sample size of the current study.

High-risk patients with nonpalpable masses should be imaged early, as was done in this study by using ultrasound. Previous studies have also identified women with BI-RADS-3 mass lesions using targeted ultrasound, which is more sensitive than screening ultrasound.^[29,30]^

Our results that 15 BC patients were diagnosed during the 1-year follow-up of the nonpalpable group (10 *in situ* and 5 invasive) show the importance of close follow-up of these patients. The results of our study show not only that these patients have higher odds of developing BC, but also that masses growing to palpability have higher odds of becoming invasive cancer. Therefore, patients with nonpalpable breast masses should be regularly assessed, and surgery should be suggested if any change in the mass occurs. Patients with a positive family history should undergo surgery.

One limitation of our study is that it focused only on breast masses, ignoring other variables associated with diagnosis in these patients. The second limitation was that patients with nonpalpable breast masses were followed up for only 1-year. With a longer follow-up, more clues related to BC can be obtained. The third limitation was that ultrasound was the only method used for cancer detection, whereas mammography could have provided additional information about BC.

In this study, we found 131 cases of BC during screening and follow-up. The calculated morbidity is 240.2/100,000, which is higher than the 10.1/100,000 observed in previous studies in China.^[1,2,3]^ However, the data in China was before 2010, and our data was from 2014 to 2015 for screening, and 2016 for follow-up. The BC morbidity has been increasing in recent years in China, and 50 patients were found during the follow-up period. Regular follow-up is essential for women with nonpalpable breast masses to prevent these masses from developing into BC.

We propose that all patients with nonpalpable breast masses and BI-RADS > 5 should undergo surgery, while those with BI-RADS > 3 should undergo surgery in case of positive family history and overweight/obesity. Finally, patients with palpable breast masses, especially those older than 50 years, with BMI > 24 kg/m^2^, and/or positive family history, should be advised surgery.

## 5 Conclusion

From the 1335 patients with nonpalpable breast masses, 15 BC cases (1.1%) were identified, including 10 *in situ* (4 overweight) and 5 invasive cancer cases (2 overweight) during the 1-year follow-up. Women with nonpalpable breast masses who are overweight/obese, more than 50 years old, and/or a positive family history are prone to develop BC and require regular clinical follow-up.

## Author contributions

**Conceptualization:** Tao Xu.

**Data curation:** Qinhua Huang, Chumiao Zhang.

**Investigation:** Qinhua Huang, Haiyan Chen.

**Methodology:** Qinhua Huang.

**Project administration:** Tao Xu.

**Resources:** Tao Xu, Chumiao Zhang.

**Software:** Chumiao Zhang.

**Supervision:** Chumiao Zhang, Haiyan Chen.

**Validation:** Haiyan Chen.

**Visualization:** Haiyan Chen.

**Writing – review & editing:** Hongmei Wen, Tao Xu, Qinhua Huang, Chumiao Zhang, Haiyan Chen.
